# Appraising Travelbee’s Human-to-Human Relationship Model

**Published:** 2016-09-01

**Authors:** Gary Shelton

Finding meaning in suffering could be one of life’s greatest quests. It is a universal question, yet we all attribute its meaning personally. Joyce Travelbee, a nurse theorist of historical significance, set about to provide the basis for such discovery. In her grand theory, the Human-to-Human Relationship, Travelbee (1971) writes: "Every human being suffers because he *is* a human being, and suffering is an intrinsic aspect of the human condition" (p 61).

To explicate the philosophic and theoretic assumptions of Travelbee’s model, and therefore ascertain its usefulness as a foundation for research, it is imperative to critically appraise this theory. An in-depth critique of the Human-to-Human Relationship Model allows an objective and nonjudgmental exploration as well as provides judgments related to the theory’s applicability (Fawcett, 2005). Through phases of her theory, including rapport, empathy, and sympathy, one establishes ways to garner the meaning of suffering (Travelbee, 1963).

As a career professional in an oncology setting, a better understanding of Travelbee’s theory should provide the advanced practice nurse (APN) an impetus and the scientific underpinnings to further nursing theory, nursing research, and evidence-based practice. This content could easily apply to all advanced practitioners as well. 

## THE HUMAN-TO-HUMAN RELATIONSHIP MODEL

**Purpose**

Joyce Travelbee believed that everything the nurse (as a human) said or did with an ill person (as a human) helped to fulfill the purpose of nursing. The nurse and the patient are human beings, relating to each other. The process is that of interaction. Nursing is an interpersonal connection, whereby the nurse facilitates the progress of a patient, a family, or a community in preventing or coping with an illness or with suffering in ways that could lead to finding meaning with the experience. The nurse is responsible for educating and providing strategies to assist the patient in avoiding or alleviating the distress of unmet needs (Pokorny, 2010; Travelbee, 1971).

Thus, the AP has an opportunity to promote human-to-human connections. This should facilitate the attribution of meaning or at least a better understanding of humans’ symptom burden and illness. By incorporating the concepts of Travelbee’s model, the AP fosters self-reflection of his or her own humanness and how an individual human relates to another. These concepts align well with the AP’s understanding of evidence-based practice and allow for developing quality improvement (QI) and nursing research.

**Concepts and Definitions**

Travelbee expresses the importance for nurses to understand their concept of what is human, for their relationship with another human being will be otherwise determined by that concept. The *human being* is defined by Travelbee, 1971 as "a unique irreplaceable individual—a one-time being in this world, like yet unlike any person who ever lived or ever will live" (p 26). Human beings are evolving; they are ever in the present but becoming. As we understand our own humanness, we grow and develop more humanness. The AP promotes patient- (or human-) centered care, which acknowledges the individuality of each human being.

Defining the concept of *patient* is a stereotype and category. Travelbee, 1971 impresses upon nurses that "actually there are no patients. There are only individual human beings in need of care, services and assistance of other human beings" (p 32). And since *nurses* are human beings, Travelbee, 1971 notes: "All assumptions about being human therefore apply to every human being categorized as nurse" (p 39).

*Illness* is a classification and category. An individual will react to illness depending on culture, symptom burden, and whether there is a related significance to those symptoms. Depending on the impairment of functioning as well as the health-care provider’s responses, a human connection that fosters understanding of the illness is developed (Travelbee, 1971). As noted, every human experiences *suffering*, as it is a part of being human. Travelbee (1971) pointed out: "It is probable that the more an individual cares for, and about others, the greater the possibilities of suffering" (p 64). Hope is future-oriented. Without hope, there is no direction for lessening suffering. Travelbee (1971) continued: "It is the role of the nurse to assist the ill person to experience hope in order to cope with the stress of illness and suffering" (p 77).

*Communication* is a necessity for good nursing and a fundamental part of this theory. Travelbee (1971) expresses striving to communicate "to know ill persons, to ascertain and meet nursing needs and to achieve the purpose of nursing" (p 102). Thus, the AP promotes the ever-evolving human-to-human connections that promote the understanding of illness and suffering.

**Relationships and Structure**

Furthering Travelbee’s assertion that we are all human beings, to be a nurse, or to be ill, the relationship is human to human. Human relationships become therapeutic as they pass through expected steps or stages. Travelbee stated (as cited by Pokorny, 2010) that nursing is accomplished beginning with "the original encounter, which progresses through stages of emerging identities, developing feelings of empathy and later, sympathy, until the nurse and the patient attain rapport" (p 61).

Mary Ellen Doona (1979) related: "A relationship is established only when each participant perceives the other as a unique human being" (p 149). The Human-to-Human Relationship is established as an interactive process. The inaugural meeting or encounter may immediately establish a connection. Unfortunately, this connection may not be positive. Through the emergence of various personal identities, both humans attempt to relate or find meaning in their encounter. Through our existence, we find meaning that creates who we are. Our uniqueness is defined by our perceptions of self and other.

As humans share in another’s experience, one can empathize or relate to the other’s experience. Sympathy surfaces in response to a human’s desire to relieve or lessen another human’s suffering (Travelbee, 2013). Travelbee (1964) explained: "Sympathy is not a phase in the process of knowing...It is rather a predisposition, an attitude, a type of thinking and feeling characterized by deep personal interest and concern" (p 70). Sister Callista Roy (1988) noted: "Travelbee added the dimension that suffering is a common life experience and that human relationships are what help people cope with suffering. Basically, nursing is a relationship of human being to human being" (p 27).

The AP is keenly aware that suffering is not always blatant or acknowledged. As a human who understands humanness, the AP anticipates human suffering, even in silence, and promotes a therapeutic relationship that allows for the exploration of meaning. Rapport, the final phase or layer of the relationship, is established secondary to the nurse/human’s knowledge and skills necessary to facilitate lessening of another human’s suffering. The nurse/human perceives, responds, and appreciates the uniqueness of the ill human being (Travelbee, 2013, 1971, 1963; Rodin, Mackay, & Zimmerman, 2009; Pokorny, 2010). Rapport is defined by Travelbee as "a process, a happening, an experience between two persons. It may not be a mutual affair at first, but the sharing of the experience and participation in it grow as each individual unfolds him or herself in the interpersonal situation...Rapport is a dynamic, fluctuating affair and it will change as changes occur in the interpersonal situation or relationship" (Travelbee, 1963, p 70).

**Assumptions**

Central to the discipline of nursing are the four phenomena of interest: person, health, environment, and nursing—nursing’s metaparadigm. Joyce Travelbee’s Human-to-Human Theory is a conceptual framework belonging to the totality paradigm. Jacqueline Fawcett (1984) explained: "The metaparadigm of any discipline is a statement or group of statements identifying its relevant phenomena...No attempt is made to be specific or concrete at the metaparadigm level" (p 84).

*Person* is defined as being human. Nurse as well as patient, family, or community under the umbrella of illness is human. Doona (1979) relayed Travelbee’s thoughts that "A person is a contingent being to whom things happen which are beyond his control…The person suffers and chooses. Through this search for meaning he creates himself" (p 11). Human beings are unique, irreplaceable, ever evolving, and interacting (Travelbee, 1971, 2013).

*Health* is defined as being both subjective and objective. Human beings perceive and relate their own sense of health and illness. To be human is to experience illness. Travelbee (1971) wrote: "A basic assumption is that illness and suffering are spiritual encounters as well as emotional-physical experiences" (p 61). Humans may see illness as having merit or as unavoidable. The presence of distress may not cause one to seek help (Travelbee, 1971, 2013).

*Environment* is not well defined, which one might relate to the timing of Travelbee’s writing, the 1960s. Instead, Travelbee relates that the nurse must be observant of the patient in the place where the patient is present in order to ascertain that the patient is in need. She speaks of experiences encountered by all humans: suffering, pain, illness, and hope. Her work with psychiatric patients and community as well as hospitalized individuals encompass an awareness of differing environments (Travelbee, 1971, 2013; Doona, 1979).

*Nursing* is better defined. Foremost, the assumption of nursing is to establish a human-to-human relationship. Doona (1979) explained: "A relationship is established only when each participant perceives the other as a unique human being" (p 149). It is within the paradigm of nursing that the nurse/human facilitates the individual, family, or community to prevent or cope with illness and suffering. The nurse also assists with trying to find meaning in these experiences (Travelbee, 1971, 2013; Pokorny, 2010). All contact with ill persons helps fulfill the purpose of nursing. Travelbee (1971) insisted: "The final measure of nursing competency is always in terms of the extent to which individuals and families have been assisted with the problems of illness and suffering" (p 119).

One could debate that in an oncology setting, there would be no difference between treating cancers as chronic diseases than treating illness in a primary care setting, except the triggers of distress occur more often. For the individual or family facing a cancer diagnosis, even if the treatment is successful, there remain an ongoing evaluation through scans and a diagnostic workup, which encourage distress and suffering secondary to the anticipation of progression of disease.

The concept of communication resonates through Travelbee’s model. Getting to know another human being is as important as performing procedures. As noted, the nurse must establish a rapport, otherwise he or she will not know the patient’s needs. Travelbee’s model is useful in this setting. Travelbee (1971) noted: "Nurses who know ill persons are more apt to be able to detect not only obvious changes in an individual’s condition but are enabled to recognize the more subtle changes that may be occurring" (p 98). The AP in the oncology setting will be able to anticipate an individual or family member’s likelihood of distress.

## The Critical Appraisal

Conceptual frameworks are constructs joined together as a basis to form a new theory. The analysis and evaluation of theory involve objective descriptions and judgments about the extent to which theories meet certain criteria (Fawcett, 1995, 2005). Since the understanding of nursing theory changes as it is analyzed and tested, it is helpful to critically appraise concepts and constructs, creating a framework upon which to further build. The explication of theory is a critical and necessary process that is both empiric and aesthetic, thus allowing for alternative opportunities to find scientific truth.

**Clarity or Brilliance**

Although complicated and layered in definitions, Travelbee’s theory clearly outlines the steps to understanding her concepts. Various sources (Travelbee, 2013) report a vague interpretation for defining her theory, but she clearly defines the concept of suffering, hope, illness, and the steps or phases necessary to establish a rapport (Travelbee, 1971). The challenge for nurses is to identify themselves as being individually human, as are their patients, and therefore accept and understand each other’s perceptions of self and illness, striving to know each other and meet each other’s needs.

**Simplicity or Parsimony**

If the Human-to-Human Relationship Theory were merely to account for nurses and patients being both human, and therefore able to relate on an equal playing field, Travelbee’s theory would appear simply stated and parsimonious. This is not the case. Multiple variables exist to define our being human, thus separating us via the level of distress and suffering. How humans define or accept their distress and suffering is multifaceted.

The AP is ever aware of an individual human’s culture, religion, ethnicity, family, and community connections, or lack thereof, and should identify ways to connect human to human. Although her theory’s simple goal is to establish a rapport with ill human beings, there are several phases or stages to accomplish: encounter, identity, empathy, sympathy, and rapport (Travelbee, 1971).

**Generalizability**

The Human-to-Human Relationship Theory has the potential for global use within nursing, as we are all human, we all have distress, and we all suffer. However, the individual human, family, or community must see his or her distress or illness as being in need of an intervention if a relationship is to develop. Spiritual values may determine one’s perception of illness or distress. Travelbee (1971) related: "The spiritual values of the nurse or her philosophical beliefs about illness and suffering will determine the extent to which she will be able to help" (p 16).

**Accessibility**

At quick glance, this theory defines concepts but does not have operational definitions for empiric research. Travelbee’s language is existential and requires an understanding of one’s perceptions of illness and suffering to find meaning. The descriptive structure of this theory is more concrete than its process. Although Travelbee’s theory lacks simplicity, her language and rhetoric can reach researchers and practitioners in human science, thus creating the foundation for generating knowledge.

**Importance**

Travelbee provides nursing with the criteria for connecting to ill persons. She has created a conceptual framework upon which to base therapeutic relationships with patients, families, and communities in distress or having the potential for suffering. Her definitions of the components of the metaparadigm of nursing’s phenomena of interest add to the social significance and social utility of her theory (Roy, 1988). Travelbee’s model teaches nurses to understand—or at least explore—the meaning of illness and suffering in themselves. It is through this existential identification that one human being can relate to another human being. The AP should promote self-reflection as human to help other humans connect.

**Theory Applications**

Travelbee’s Human-to-Human Relationship Theory that patients are seen as unique individuals and as human beings is in keeping with the current guidelines and expectations set forth by agencies such as the Institute of Medicine, the American Nurses Association, and the Joint Commission for Hospital Accreditation. Care should be patient-centered. The theory is applicable to and has been used in the hospice movement, helping terminally ill individuals and their families find meaning in suffering and fostering hope, even at end of life (Herth, 1990). Margaret Moses (1994) explored Travelbee’s concern over nursing care’s lack of compassion: "An individual’s interpretation of caring affects the quality of care they [sic] can provide" (p 202).

## Conclusion

Travelbee’s grand theory of Human-to-Human Relationships provides nurses with a foundation necessary to connect therapeutically with other human beings. The assumptions involve humans, who are nurses, relating to humans who are suffering, are in distress, or have the potential to suffer. Travelbee stated (as cited in Reed, 1992): "Experiencing meaning in illness, in particular, has long been identified as an important clinical phenomenon" (p 354). Because of the nurse’s knowledge and experience, he or she develops a rapport with ill humans. Nurses perceive and understand the uniqueness of every ill human being and therefore facilitate their finding meaning in suffering (Travelbee, 2013). The AP has an opportunity to promote human-to-human connections. This should facilitate the attribution of meaning or at least a better understanding of humans’symptom burden and illness.
